# Supplementary material for: Literature-based latitudinal distribution and possible range shifts of two US east coast dune grass species (Uniola paniculata and Ammophila breviligulata)
Source: PeerJ. 2018 Jun 8;6:e4932. doi: 10.7717/peerj.4932 (PMC5996817; doi:10.7717/peerj.4932)
Supplement: Supplemental Information 1 [file peerj-06-4932-s001.docx]

# **Supplemental S1**

*(These are the methods that were distributed to each author at the beginning of the data collection):*

General info

-’Publications’ refer to previous published work. They exist in the ‘Papers’ folder in this Google Drive

-The ‘Manuscript’ here refers to our work, it also in the Google Drive

Goal:

-’Exhaustive’ search of literature

-Spreadsheet populated with geographic locations of UNPA and AMBR. studies.

-Make a Map

-Add text to the Manuscript file

Demarcation— we are focused on:

1. Statements regarding the Northern range limit of UNPA
2. Statement regarding the Southern range limit of A.brev.
3. Studies focusing on these species (in a Dune context) from NJ to NC.
4. Occurrence in the field (from NJ to NC) that is listed or described in a paper/database for these two species.
5. Greenhouse and Lab studies focusing on UNPA and AMBR that relate to climate and range. These will not have a map location, but should be mentioned in the discussion section of the paper.

Map Workflow:

1. In the ‘Papers’ folder of the Google Drive, pick a publication that has no ‘Star’ next to it
2. Rename the file and place an asterisk [*] at the beginning of the file name to show that you are ‘claiming’ it.
3. Read/skim the publication. There are two types of information we are interested in for the map. **First**, any comments about species range made by the author (i.e., not comments about range that are based on other referenced sources). **Second**, any work done by the author on UNPA or AMBR at specific sites.
4. Is there is a comment about **Species Range Limit** from the author? If so, put info in the Google Sheet
   1. Location (1-2 word description e.g. “South Core”, “Cape Henry”)
   2. Add the state
   3. The in text citation (i.e., ‘Woodhouse et al., 1977’)
   4. Add the year (seems redundant, but will make life easier)
   5. Add appropriate label for species (‘A’ or ‘U’)
   6. Add ‘R’ in obs_type column for range limit
   7. Coordinates of the study site(s) — (lat/lon in decimal degrees). Many times the coordinates or precise location are not specified, and you, as the person reading the paper and filing in the document, will need to pick the coordinates. If you picked the coordinates, put a ‘1’ in the ‘selectedbyreader’ column, otherwise put a ‘0’ if the paper defined the coordinates or a specific study site.
   8. DOI defined as a link — i.e. http://doi.org/10.2307/1948464
   9. Add the name of the journal
   10. In the ‘Notes’ column of the spreadsheet, identify where in the manuscript you founds the information (i.e., ‘page six, third line of paragraph 2’)
5. Is there is a study done by the author, or a collection or observation made by the author about AMBR or UNPA? If so, put info in the Google Sheet
   1. Location (1-2 word description e.g. “South Core”, “Cape Henry”)
   2. Add the state
   3. The in text citation (i.e., ‘Woodhouse et al., 1977’)
   4. Add the year (seems redundant, but will make life easier)
   5. Add appropriate label for species (‘A’ or ‘U’)
   6. In the next column (obs_type) put a ‘P’ (for papers where the grass only exists because it was planted by author as part of the study) or ‘E’ (Existing))
   7. Coordinates of the study site(s) — (lat/lon in decimal degrees). Many times the coordinates or precise location are not specified, and you, as the person reading the paper and filing in the document, will need to pick the coordinates. If you picked the coordinates, put a ‘1’ in the ‘selectedbyreader’ column, otherwise put a ‘0’ if the paper defined the coordinates or a specific study site.
   8. DOI defined as a link — i.e. http://doi.org/10.2307/1948464
   9. Add the name of the journal
   10. In the ‘Notes’ column of the spreadsheet, identify where in the manuscript you founds the information (i.e., ‘page six, third line of paragraph 2’)
6. Put the full reference of the publication in the manuscript
7. Snowball Sampling: Are there other relevant works mentioned in the paper? Are they missing from the ‘Papers’ folder? Does your paper get cited by other relevant literature (i.e., using WoS or Google Scholar) that is not included in the ‘Papers’ folder? Check to see if the paper is already in the ‘Papers’ folder. If not, find those pdfs and place them in the ‘Papers’ folder. If you cannot find a pdf/reference, then cut and paste the reference into the Google doc entitled ‘Missing Refs’ inside the ‘Papers’ folder
8. Rinse and Repeat — go back to Step 1

Map Example:

An example comes from Wagner 1964. Wagner studied UNPA on Bogue Banks, so there will be an entry in the spreadsheet for ‘Bogue Banks’ with a ‘U’ label for UNPA, an ‘E’ label for existing prior to the work (even though Wagner did both in situ and transplant experiments, UNPA was present at the site prior to work). The appropriate DOI and coordinates (for Bogue Banks) would also be put in the google sheet, and the full text citation would go in the manuscript.

In addition, Wagner (1964) also comments on UNPA’s northern range limit:


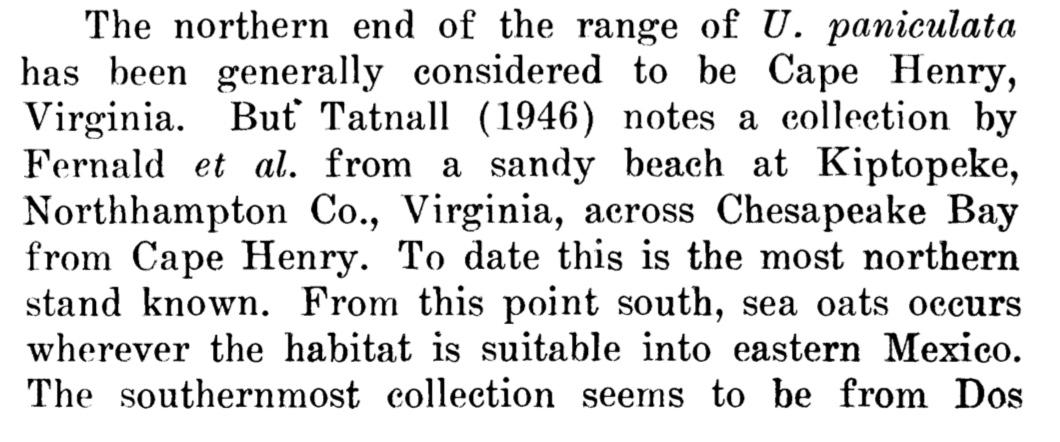


So this deserves an entry in the spreadsheet for ‘Cape Henry’ with a ‘U’ label for UNPA, an ‘R’ label for range limit. The appropriate DOI and coordinates (for Cape Henry) would also be put in the google sheet, and the full text citation would go in the manuscript.

As a result, Wagner (1964) will have two entries on the spreadsheet. In addition, when you see a paragraph like this, the Tatnall (1946) reference should be hunted down, placed in the Google Drive folder (so it can be read and imported into the dataset and placed on the map)...

Greenhouse/Lab studies

Some studies may focus on lab or greenhouse work with UNPA or AMBR. These studies do not have a map location, but if they are relevant in the context of climate change/ range, then they should be mentioned in the discussion. So if you come across studies, comments, text that is relevant (i.e., mentions of mechanistic controls on range limit), then please add both text and a citation to the manuscript file.
