# Supplementary material for: Literature-based latitudinal distribution and possible range shifts of two US east coast dune grass species (Uniola paniculata and Ammophila breviligulata)
Source: PeerJ. 2018 Jun 8;6:e4932. doi: 10.7717/peerj.4932 (PMC5996817; doi:10.7717/peerj.4932)
Supplement: Supplemental Information 2 [file peerj-06-4932-s002.doc]

**
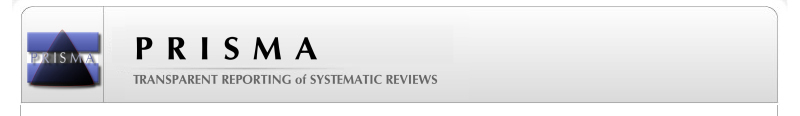
PRISMA 2009 Flow Diagram**

**Screening**

**Included**

**Eligibility**

**Identification**

Records identified through database searching (WOS, GS)
(n = A = 97)

Notes:

A = total papers (manuscript) (97)

B = papers that made comments about range that were too broad (1) — Tatnall 1946

GBIF records are not included here.

Additional records identified through other sources (GBIF)
(n = B: 1738);

Records after duplicates removed
(n = A = 97)

Records screened
(n = A = 97)

Records excluded
(n=0)

Full-text articles assessed for eligibility
(n = 97)

Full-text articles excluded, with reasons (n = B= 1; Tatnall 1946, too broad)

Studies included in qualitative synthesis
(n = A-B= 96)

Studies included in quantitative synthesis (meta-analysis)
(n = A-B= 96)
