# Supplementary material for: Literature-based latitudinal distribution and possible range shifts of two US east coast dune grass species (Uniola paniculata and Ammophila breviligulata)
Source: PeerJ. 2018 Jun 8;6:e4932. doi: 10.7717/peerj.4932 (PMC5996817; doi:10.7717/peerj.4932)
Supplement: Supplemental Information 3 [file peerj-06-4932-s003.html]

leaflet

 
